# Combinations of Calcitriol with Anticancer Treatments for Breast Cancer: An Update

**DOI:** 10.3390/ijms222312741

**Published:** 2021-11-25

**Authors:** Mariana Segovia-Mendoza, Janice García-Quiroz, Lorenza Díaz, Rocío García-Becerra

**Affiliations:** 1 Departamento de Farmacología, Facultad de Medicina, Universidad Nacional Autónoma de México, Ciudad de México 04510, Mexico; mariana.segovia@facmed.unam.mx; 2 Departamento de Biología de la Reproducción Dr. Carlos Gual Castro, Instituto Nacional de Ciencias Médicas y Nutrición Salvador Zubirán, Vasco de Quiroga No. 15, Belisario Domínguez Sección XVI, Tlalpan, Ciudad de México 14080, Mexico; janice.garciaq@incmnsz.mx; 3 Departamento de Biología Molecular y Biotecnología, Instituto de Investigaciones Biomédicas, Universidad Nacional Autónoma de México, Ciudad de México 04510, Mexico

**Keywords:** breast cancer, calcitriol, drug combination, efficacy

## Abstract

Preclinical, clinical, and epidemiological studies indicate that vitamin D3 (VD) deficiency is a risk factor for the development of breast cancer. Underlying mechanisms include the ability of calcitriol to induce cell differentiation, inhibit oncogenes expression, and modify different signaling pathways involved in the control of cell proliferation. In addition, calcitriol combined with different kinds of antineoplastic drugs has been demonstrated to enhance their beneficial effects in an additive or synergistic fashion. However, a recognized adjuvant regimen based on calcitriol for treating patients with breast cancer has not yet been fully established. Accordingly, in the present work, we review and discuss the preclinical and clinical studies about the combination of calcitriol with different oncological drugs, aiming to emphasize its main therapeutic benefits and opportunities for the treatment of this pathology.

## 1. Introduction

### 1.1. Vitamin D Metabolism

Vitamin D (VD) is a generic name encompassing different lipidic metabolites derived from 7-dehydrocholesterol in animals or ergosterol in fungi and plants. These metabolites are considered secosteroids due to the breakage of the B ring of the cyclopentanoperhydrophenanthrene structure by sunlight exposure. Examples of these naturally occurring secosteroids include lumisterol (VD1), ergosterol (VD2), cholecalciferol (VD3), calcidiol (25-hydroxyvitamin D2), and calcitriol (1,25-dihydroxyvitamin D3). Calcitriol represents the most active VD metabolite and hormonal form, which modulates calcium homeostasis through actions on the kidney, bone, and intestinal tract [1]. VD3 is formed in the skin after a complex series of steps. The process begins with the photoisomerization of 7-dehydrocolesterol to pre-VD3 under the influence of UV B radiation (wavelength, 280–315 nm). Once formed in the skin, VD3 is transported in the blood by the VD binding protein (DBP) and can be 25-hydroxylated in the liver to calcidiol by the mitochondrial and microsomal enzymes CYP27A1 and CYP2R1, respectively. Afterward, this intermediate metabolite may be activated by CYP27B1 in the kidney, producing calcitriol. This hormone enters into the cells by passive diffusion and binds to the intracellular vitamin D receptor (VDR), forming a complex with the retinoid-X receptor (RXR) [2]. The resulting heterodimer binds to vitamin D response elements (VDREs) sequences in the DNA to either promote or suppress the gene expression, depending on the type of co-activators or co-repressors recruited.

VDR is expressed in several tissues and cells; it has a higher affinity towards calcitriol than any other VD metabolite. Among the effects that this hormone induces in cancer cells are the arrest of the cell cycle, associated with cyclin-dependent kinases (CDK) inhibition, and apoptosis (modulation of Bcl-2 protein family) [3,4,5,6,7]. In addition, calcitriol can also activate different non-genomic actions such as protein kinase C, modulate phospholipid metabolism, stimulate the formation of cyclic nucleotides, trigger calcium transport, and regulate Raf and mitogen-activated protein kinase (MAPK) extracellular signal-regulated kinases (ERK) signaling, all in a manner independent of VDR/DNA binding [6,8,9,10,11,12].

Additionally, calcitriol or its different synthetic analogs (e.g., alfacalcidol, paracalcitol) inhibit cancer cell proliferation [13,14]. The mechanisms so far reported by which calcitriol acts in cancer cells are diverse. Standing out among these are the promotion of cell differentiation, anti-inflammatory effects, apoptotic actions, among others [15,16,17]. Diverse preclinical and clinical studies have focused on studying combinations of calcitriol with a variety of agents with and without chemotherapeutic action in different cancer cells, including breast cancer [18,19,20,21,22]. Unfortunately, the use of calcitriol or its analogs in cancer treatment as single agents or in combination with other antineoplastic compounds has been hindered by the failure to achieve outstanding outcomes. However, its possible application in the clinic is being actively studied, and several approaches may be considered, including optimal and intermittent doses of therapy, appropriate clinical regimens, and efficient selection of patients who could benefit of this strategy, as was previously discussed by Trump in his recent review [23].

### 1.2. Breast Cancer Disease and VD

Breast cancer (BC) is the most frequent neoplasm in women around the world [24]. This disease can be mainly divided into three different types according to the expression of specific molecular targets. The most common BC subtypes are estrogen receptor (ER) positive, epidermal growth factor receptor type 2 (HER2) -positive, and triple-negative (TNBC) [25,26]. Among these, the ER subtype is commonly found in around 70% of BC cases. It has been linked with a better prognosis when compared with other BC varieties [27]. Noteworthy, calcitriol has an essential role in normal mammary gland development. Animal models based on VDR knockout mice have confirmed this assumption, as alterations in mammary morphogenesis have been observed, as well as increased growth, in response to exogenous estrogen and progesterone compared to wild-type mice [28,29].

Epidemiological studies have pointed out a close relationship between VD3 deficiency and the development of BC. Different studies have also correlated higher serum calcitriol levels in the early stage of BC compared to more advanced stages and metastatic progression of this disease. VD3 deficiency is diagnosed when serum calcidiol levels are below 20 ng/mL (50 nmol/L) [30]. In addition, not only have low levels of calcidiol been associated with the development of BC, but deregulated components of the VD3 biosynthetic pathway and altered VD3 transcriptional functions also participate in the development and establishment of this pathology. For instance, the aberrant amplification of the *CYP24A1* gene in BC has been reported. This gene encodes the 24-hydroxylase enzyme, which is responsible for calcitriol degradation. Therefore, CYP24A1 overexpression could lead to abrogation of growth control mediated by calcitriol [31,32]. On the other hand, CYP27B1, responsible for calcidiol activation, has been detected in normal human breast as well as in breast carcinoma samples [33], indicating that normal and malignant breast tissue can locally synthesize the active form of VD3. However, CYP27B1 degradation has been found enhanced in tumors, precluding the antineoplastic effects of calcitriol. In addition, the expression of VDR has been reported in normal and malignant BC cells, corroborating that transformed cells are also under the control of VD metabolites [34]. Of note, the loss of VDR has a critical impact on the survival of patients with BC [35].

The antineoplastic effects of calcitriol or its different analogs administered alone or in combination with chemotherapeutics agents have also been widely reported in in vitro and in vivo models using different BC cells. Nevertheless, the optimal response depends on different factors, including molecular type and stage of BC, kind of therapy, the status of VDR, among others [36,37,38].

In the section below, we will describe the types of therapeutic agents that have been combined with calcitriol taking into consideration their mechanism of action in BC, to lay the bases that can assist for the establishment of calcitriol as an adjuvant agent in this pathology.

## 2. Calcitriol in Combination with Chemo/Radiotherapy in BC

Most cancer cells can respond to calcitriol by expressing the VDR. In particular, BC cells have shown higher VDR protein levels compared to benign breast tissue [39]. Through this transcription factor, calcitriol exerts many anticancer effects, including growth inhibition, induction of cell differentiation, anti-inflammatory activity, cell cycle arrest, oncogenes downregulation, and many others that place calcitriol as a natural endogenous cancer-preventive antineoplastic factor [40,41]. This has been the basis of the vast number of studies designed to study calcitriol and its analogs as pharmacological options in the oncological setting [42]. Notably, calcitriol not only acts as an antineoplastic agent, but also can help to overcome drug resistance, increase the susceptibility to chemotherapy and even potentiate the effects of conventional chemotherapeutic agents and radiation therapy. We will address this last subject in this section, focusing on BC.

A vast number of preclinical studies has explored the potential enhancement of calcitriol anticancer effects by its combination with conventional chemotherapeutic regimens. This, of course, has the additional benefit of allowing for dose reduction of the chemotherapeutic drug, while at the same time minimizing unwanted side effects.

### 2.1. Enhancement of BC Responsiveness to Chemotherapeutic Agents and Radiation by Calcitriol

TNBC tumors are challenging to treat since they do not express druggable targets such as ER, progesterone receptor (PR), or HER2, precluding a tailored therapy. Therefore, TNBC tumors are preferentially treated with chemotherapeutic agents such as platinum compounds (e.g., cisplatin, carboplatin), taxanes (e.g., paclitaxel, docetaxel), anthracyclines (e.g., doxorubicin, epirubicin), antimetabolites (e.g., 5-fluorouracil, methotrexate), alkylating agents (e.g., cyclophosphamide) or their combinations [43,44]. Interestingly, it has been described that approximately one-third of TNBCs express the VDR, which inversely correlates with the mitotic score, histological grade, proliferation index, and recurrence [45]. At the same time, patients with VDR-positive tumors have shown more prolonged overall survival (26 months) than VDR-negative ones [45]. More recently, RNA-sequencing data analysis of different basal-like patient-derived xenografts has shown that one of the most highly expressed genes in TNBC is the VDR [46]. Notably, other BC subtypes such as ER-positive, PR-positive, and HER2-positive also express VDR in a high percentage of tumor cells, a feature found to be associated with more favorable prognostic characteristics and less aggressive phenotypes [47]. The choice of treatment in each of these cases is based on tumor histopathological features, molecular markers and clinical characteristics, and may include chemotherapy in conjunction with a targeted therapy [48,49]. Therefore, given the high rate of VDR expression in BC tumors, it is feasible to target this receptor in conjunction with chemotherapeutic drugs in the different BC subtypes. In this regard, more than 20 years ago, Koshizuka and colleagues demonstrated the ability of three VD compounds to enhance paclitaxel antineoplastic effects in vivo in BC. They showed that calcitriol and two of its analogs, EB1089 and 1,25(OH)2-16-ene-23-yne-19-nor-26,27-F6-D3, produced greater antitumor activity than paclitaxel alone and exerted an additive effect when administered with the taxane. EB1089 was the most potent compound by itself and the one to produce the most active antitumorigenic combination, which was promising considering that EB1089 is a non-calcemic calcitriol analog [50]. The same laboratory obtained similar results with another VD analog: CB1093, which enhanced paclitaxel antitumor activity in mice carrying MCF-7 xenografts. Cisplatin was also tested with this VD analog, but the results were greater using the taxane [51]. These studies were undertaken using a luminal A-type BC cell line, which is ER-positive. However, similar results have also been demonstrated in TNBC cells and other ER-positive cell lines. Notably, Wang et al. showed that pretreating MDA-MB-231, MCF-7, and T-47D cells with calcitriol before exposing them to paclitaxel or doxorubicin, decreased the half-maximal inhibitory concentration (IC50) by up to 2 logs for paclitaxel and up to 1 log for doxorubicin, when considering as endpoints colony formation inhibition and cell death induction. The mechanism behind these effects resulted to be apoptosis, and in the case of paclitaxel, the pretreatment of cells with calcitriol improved the taxane-dependent B cell CLL/lymphoma-2 (Bcl-2) phosphorylation [52]. Noteworthy, the co-treatment was more effective in ER-positive cells compared to TNBC cells. However, and very interesting, Wilhelm and colleagues showed that the effect of calcitriol or its precursor calcidiol on paclitaxel efficacy differed within the TNBC subtypes and depended on p53-positivity and VDR status [53].

More recently, Klopotowska and Matuszyk compared the ability of calcitriol and its analog tacalcitol to improve the antineoplastic activity of 5-fluorouracil in different BC cell lines representing various molecular subtypes. They found that among the six BC cell lines tested, the VDR agonists more efficiently enhanced 5-fluorouracil anticancer activity in the luminal subtypes [54]; thus, showing similar results to those just described with calcitriol and paclitaxel. In accordance, other studies have shown that calcitriol, by inducing metabolic reprogramming, renders BC cells more susceptible to chemotherapy. In particular, the addition of calcitriol to MCF-7 cells was found to improve 5-fluorouracil and CBR-5884 antiproliferative effects significantly [55]. Of note, CBR-5884 is a phosphoglycerate dehydrogenase inhibitor that decreases de novo serine synthesis in cancer cells.

One major drawback of chemotherapy is the possibility of metastasis formation. In a recent preclinical study, Zheng and colleagues described a methodology to deliver paclitaxel along with calcitriol by pH-sensitive micelles, allowing for an efficient tumor uptake of the paclitaxel+calcitriol combination. This treatment suppressed primary tumor growth and inhibited lung metastasis formation in 4T1 tumor-bearing mice as a result of matrix metalloproteinase-9 (MMP)-9 and Bcl-2 level downregulation as well as E-cadherin upregulation [56]. 4T1 is an animal model for stage IV human BC. The authors suggested the possibility to translate this finding into the clinic to counteract the pro-metastatic effect of paclitaxel in TNBC therapy.

Cancer stem cells (CSCs) have unlimited potential for self-renewal, they are able to drive tumorigenesis and may give rise to a phenotypically diverse progeny resulting in tumor heterogeneity with differential sensitivity to chemotherapeutic agents [57]. Specifically, BC stem cells (BCSC) are implicated in cancer recurrence, tumor initiation and progression, distant metastasis, and resistance to therapy. Regarding this, CSCs are known to be less sensitive to chemotherapy, thus, remaining viable after treatment rounds. Considering all of this, it is of utmost importance to target BCSCs in order to avoid tumor resurgence. BCSCs are known to express some distinctive cell surface markers, such as aldehyde dehydrogenase 1 (ALDH1), cluster of differentiation (CD) 44 (CD44), CD133, CD49f, CD24, and others which are commonly associated with chemotherapy and radiotherapy resistance [58]. A higher expression of these markers is associated with increased resistance to treatment and poor prognosis, as in TNBC cells [59,60]. In this regard, it has been shown that calcitriol and the VD analog BXL0124 can suppress ductal carcinoma progression in vivo and inhibit cancer stem-like cells in mammospheres [61]. In addition, these compounds have also been shown to inhibit BCSCs enrichment by inducing their differentiation, which was accompanied by the reduction of key markers of pluripotency and CSC-like phenotype in TNBC [62]. This ability of calcitriol has been exploited to sensitize BC cells to chemotherapy, opening the possibility to use it as an adjuvant treatment in cancer patients. Indeed, a study using BC in in vitro and in vivo approaches recently showed that combining calcitriol (100 nM) with cisplatin, methotrexate, or doxorubicin significantly diminished their IC50. This interaction resulted in a synergic inhibition (combination index value < 1.0) of cell proliferation and resulted in cell cycle arrest at the G2/M phase. Mechanistically, both in vitro and in vivo outcomes showed that the co-treatment significantly decreased ALDH1 levels (35–47%, depending on the chemotherapeutic agent) in MCF-7 and MDA-MB-231 cells and tumor tissues, suggesting the targeting of BCSCs by the combination, which resulted in necrosis and tumor growth inhibition [18]. The same research group has also shown that calcitriol enhanced the cytotoxic effect of paclitaxel in MCF-7 BC cells and murine mammary adenocarcinoma in vivo, which correlated with ALDH1 [63]. Similarly, using MMTV-Wnt1 mammary tumors (expressing both ER and VDR) in a murine model, it was shown that calcitriol was able to inhibit BCSCs self-renewal and tumor spheroid formation dose-dependently and that the combination of calcitriol with ionizing radiation inhibited spheroid formation to a greater extent than either treatment alone [64]. It was suggested that the Wnt/β-catenin pathway was implicated in this effect, which is important given the well-known overactivation of the Wnt/β-catenin pathway in TNBC and its correlation with poor survival [65].

### 2.2. Mechanism of Action of Calcitriol and Its Analogs to Potentiate the Response to Ionizing Radiation in BC Cells

Various mechanisms of action behind the enhancing properties of calcitriol upon radiation therapy in BC cells have been described. Here, we discuss some of them.

(a) Induction of cytotoxic autophagy. In MCF-7, HER2-overexpressing, p53 wild-type ER-positive ZR-75-1 and p53 mutant Hs578t cells (a breast tumor cell line intrinsically radioresistant), calcitriol and its analog EB1089 have been shown to sensitize cells to radiation by promoting cytotoxic autophagy. In particular, calcitriol inhibited the ability of the cells to recover after radiation. However, in BT-474 cells expressing low VDR levels, cytotoxic autophagy was not induced by calcitriol; thus, radiosensitivity was not modified by this hormone. Notably, radiation alone reduced MCF-7 colony formation by 74%, while calcitriol in combination with radiation further reduced this parameter in 87% [66,67,68,69].

(b) Reduction of RelB. RelB, a subunit of the nuclear factor-κβ (NFκβ), is known to be expressed at high levels in aggressive BC, especially in TNBC, resulting in the induction of Bcl-2 and cyclin D1 (CCND1) expression as well as promotion of cell cycle progression and cell proliferation [70]. Notably, RelB expression in BC cells also confers resistance to gamma radiation. Interestingly, one way by which calcitriol improves the prognosis of patients with BC is by inhibiting RelB expression, with in turn downregulates Bcl-2 and increases BC cell sensitivity to gamma-irradiation, as shown in Hs578T and HER2 overexpressing NF639 BC cells [71].

(c) Sensitization of BCSCs to ionizing radiation through the inhibition of the Wnt/β-catenin signaling pathway. As mentioned before, the Wnt/β-catenin signaling pathway is inhibited by calcitriol in BCSCs cells, a process that was associated with the potentiation of a clinically relevant dose of ionizing irradiation (2 Gy) to inhibit BCSC-spheroid formation [64]. 

(d) Enhancement of the antiproliferative and apoptotic effects of ionizing radiation.

The co-administration of fractionated radiation with the VD analog ILX-23-7553 has shown to exert an additive pro-apoptotic effect as well as a preventive recovery effect in MCF-7 cells. Notably, this outcome had no impact on non-malignant control cells. ILX-23-7553 has been shown to be able to increase MCF-7 cells sensitivity to radiation as much as four-fold times [64]. EB1089 has also been shown to increase BC cells sensitivity to radiation in vitro and in vivo by promoting apoptosis and increasing radiation-dependent DNA fragmentation. Importantly, this treatment had no apoptotic effect in healthy non-cancerous cells, further highlighting the feasibility to translate this therapeutic scheme to the clinic [72].

A good review on the utilization of calcitriol and its analogs as radiosensitizers in different cell lines, including the various schemes of radiation dose, mode of delivery, and radiation type, has been recently published [73].

In conclusion, calcitriol can sensitize/potentiate chemotherapeutic drugs antitumorigenic effects. Similarly, calcitriol may improve the impact of radiation on BC therapy. Thus, oncologists should consider recommending patients to assess their VD levels and supplement accordingly in the case of deficiency/insufficiency, as well as before and during chemotherapy and radiation treatment.

## 3. Combined Antitumoral Effect of Calcitriol with Natural Compounds in BC

The conventional cytotoxic agents commonly used to treat BC may cause significant unwanted side effects. An alternative to avoid the above inconvenience could be the establishment of different therapeutic strategies that involve drug combinations, targeting in this way different signaling pathways that could be used by the neoplastic cells to escape the treatment. In this regard, calcitriol, a negative growth regulator of BC cells, represents an alternative treatment approach for human cancer. Substantial evidence supports that calcitriol antineoplastic effects may be increased by its concomitant use with naturally derived compounds [63,74,75,76], including vitamins [77,78], fatty acids [79], and anti-inflammatory compounds [21,80,81,82,83], which are described below.

Natural compounds exert protective effects against cancer due to the presence of phytochemicals that act via different mechanisms of action. Calcitriol has been extensively evaluated in combination with several natural agents in leukemia [84,85,86,87]. Regarding BC, there are only a few studies, including combinations with resveratrol, curcumin, melatonin, and genistein [63,74,75,76].

### 3.1. Increased Antitumoral Effect of Calcitriol Combined with Resveratrol in BC

Resveratrol, a natural compound present in medicinal plants, peanuts, grapes, and mulberries [88], has many biological activities, including regulation of lipid metabolism, inhibition of platelet aggregation, prevention of cardiovascular disease, and hepato-protection. In addition, resveratrol has antioxidant, antimutagenic, anti-inflammatory, and antitumoral properties [89]. Regarding the last point, the antitumoral effect of resveratrol has been evaluated both in vitro and in vivo in several neoplasms, and it has been determined that this compound intervenes in the three stages of carcinogenesis: initiation, promotion, and progression [89]. In BC cell lines, resveratrol inhibits cell proliferation independently of the cancer cell phenotype [90,91,92]. In ER-positive cells, resveratrol exerts antiestrogen actions, triggering parallel pathways that counteract the cellular outcomes induced by estrogens [91]. In the ER-positive MCF-7 and ER-negative MDA-MB-468 BC cell lines, resveratrol has been shown to inhibit proliferation in a dose-dependent manner, altering autocrine growth modulator pathways [92]. In MDA-MB-231 TNBC cells, part of the mechanisms involved in the cell proliferation and migration inhibition by resveratrol includes decreasing the expression and secretion of MMP-2 and MMP-9, and reversing the transforming growth factor-beta 1 (TGF-β1)-induced epithelial-mesenchymal transition (EMT), possibly through the phosphatidylinositol 3-kinase (PI3K)/protein kinase B (AKT) signaling pathway. This activity has also been observed in vivo in mice bearing MDA-MB-231 xenografts, where resveratrol inhibited tumor growth and lung metastasis [93]. Likewise, a natural methoxylated resveratrol analog has shown similar effects, inhibiting the proliferation, invasion, and migration of MCF-7 cells by down-regulating the PI3K/AKT and Wnt/β-catenin signaling pathways [94]. Additionally, resveratrol and its analogs revert EMT in tumors [95]. In T-47D BC cells, resveratrol has been shown to induce apoptotic cell death via caspase activation, CD95 ligand expression enhancement, and induction of CD95 signaling-dependent cell death, which initiates apoptosis [88]. The antitumoral effect of resveratrol has been studied in conjoint with other antineoplastic agents in BC, including tamoxifen [96], and calcitriol [74]. In this regard, resveratrol per se has been shown to inhibit the proliferation of the TNBC cell line MBCDF-Tum in a dose-dependent manner, while this effect was potentiated by calcitriol. In vivo, the concomitant administration of resveratrol with calcitriol to mice bearing triple-negative breast tumor xenografts inhibited tumor neo-angiogenesis significantly and to a greater extent than each drug alone [74]. A possible mechanism behind these effects is the enhancement of calcitriol signaling by resveratrol, which was reported to occur at nanomolar concentrations mediated by a resveratrol-dependent VDR expression stimulation in ER-postivie BC cells [97]. This data support that dietary resveratrol sensitizes BC cells to the antiproliferative effects of calcitriol.

### 3.2. Combined Antitumoral Effect of Calcitriol and Curcumin in BC

Curcumin, a polyphenol derived from turmeric, a traditional Indian spice, has been reported as an antioxidant, anti-inflammatory, anticancer, and chemo-preventive agent for BC [98]. This natural compound exerts its anticancer effects inhibiting cell proliferation and invasiveness, through regulation of multiple intracellular signaling pathways, including modulation of NFKβ, fatty acid synthase, insulin-like growth factor I (IGF-1) axis, ER, HER-2, epidermal growth factor receptor (EGFR), among others. Curcumin also promotes apoptosis by regulating the expression of apoptosis-related genes and proteins, inducing reactive oxygen species (ROS) accumulation, inducing cell cycle arrest, acting as an antiangiogenic and anti-invasive compound, inhibiting metastasis, and modulating microRNAs involved in oncogenesis [98]. The antineoplastic effects of curcumin have been evaluated alone and in combination with other drugs, including chemotherapeutic agents [99,100], other natural compounds [101], and calcitriol [74]. Regarding the latter, the combination of calcitriol and curcumin also has been studied in conjoint with the chemotherapeutic agent paclitaxel in MCF-7 BC cells, where the triple therapy showed synergistic cytotoxic interaction, enhanced apoptotic potential, and in vivo, reduced tumor size compared to mono-treatments [63]. Similarly, the combination of curcumin with calcitriol has shown an enhanced antiproliferative effect in cultured TNBC cells compared to each drug alone. Likewise, in vivo, the coadministration of calcitriol and curcumin significantly reduced tumor onset, tumor volume, and microvessel count, which was associated with less tumor-activated endothelium, suggesting an antiangiogenic promoting effect of the drug combination. Additionally, the co-treatment increased calcitriol bioactivity, as suggested by the renal modulation of Cyp24a1 and Cyp27b1 [74]. The above suggests that the combined treatment affects each drug metabolism, resulting in increased anticancer activity.

### 3.3. Combination of Melatonin and Calcitriol in BC

Melatonin is a hormone secreted by the pineal gland at night under normal light/dark conditions; however, this compound may also be found at widely variable concentrations in beans, leaves, and roots of medicinal plants, as well as seeds, flowers, and fruits. Therefore, melatonin from plant origin represents a significant melatonin source for humans [102]. The main functions of melatonin are to mediate dark signals, with possible implications in the control of circadian rhythmicity, seasonality, mammalian immune system modulation, blood pressure control, hemostasis, and glucose regulation [103]. Additionally, it is considered a potent antioxidant [104]. In cancer cells, melatonin inhibits cell proliferation, angiogenesis, invasiveness, and metastasis, induces differentiation, and promotes apoptosis [105,106]. Disturbance of melatonin production may influence cancer genesis and growth. Regarding BC, low levels of melatonin might be a risk factor for this neoplasm; accordingly, melatonin plasma concentrations are diminished in patients with BC [107]. The mechanisms by which melatonin exerts its antitumor actions include antiestrogenic actions such as regulation of ER expression, transactivation, and modulation of enzymes involved in the local synthesis of estrogens, as well as modulation of the cell cycle, stimulation of cell differentiation and apoptosis, suppression of telomerase activity, antioxidant effects, anti-angiogenesis, prevention of circadian disruption, inhibition of metastasis, modulation of epigenetic factors, suppression of tumor metabolism and activation of the immune system [105]. The anticancer effects of melatonin have often been observed on estrogen-responsive human BC cell lines. In MCF-7 BC cells, melatonin per se enhances p53 acetylation by down-regulating murine double minute 2 (*MDM2*) gene expression, a key regulator of p53 [76]. Notably, when melatonin is combined with VD3, a synergistic proliferation inhibition has been observed, whose mechanisms involve the activation of the TGF-β1 pathway and downregulation of both MDM2 and AKT phosphorylation [75].

### 3.4. Relationship between Genistein and VD Derivatives in BC

Genistein is one of the most important isoflavones, present mainly in soybeans, followed by legumes, fruit, nuts, and vegetables, whose intake has been associated with a lower incidence of breast and prostate cancer in Asian populations [108]. Additionally, other studies in vitro support that genistein can be considered a promising chemo-preventive agent for treating different types of cancer [109,110]. The mechanism of action of this isoflavone to inhibit cancer cell growth involves cell cycle arrest [111] and modulation of genes related to apoptosis. Specifically, genistein inhibits the activation of NFκβ and AKT signaling pathways [112,113], inhibits the topoisomerase I and II, 5α-reductase, and protein histidine kinase. In addition, genistein acts as an antioxidant and is considered a potent inhibitor of angiogenesis and metastasis [114]. In BC cells, the isoflavone decreases the stem-like cell population through the Hedgehog pathway and inhibits total HER2 protein expression and phosphorylation [115,116]. Interestingly, genistein has weak estrogenic activity and bears structural similarity to 17β-estradiol, competing with it for the ER, blocking the binding of more potent estrogens, affecting estrogen metabolism, and restoring ERα expression, thereby contributing to a favorable role in the treatment of hormone-related cancers [114,117]. To study the effects of phytoestrogens on BC cell sensitivity to VD3 compounds, Wietzke and Welsh transiently transfected a VDR promoter-luciferase construct into the ER-positive BC cells T-47D and MCF-7. In this model, genistein treatment upregulated the transcription of VDR promoter and increased VDR protein expression, suggesting the sensitization of BC cells to calcitriol by this isoflavone. Interestingly, these effects were mediated by the ER [97] and in MCF-7 cells by reducing CYP24A1 and stimulating CYP27B1 expression and activity; thus, increasing the bioavailability and reducing the catabolism of the active hormone [118]. This might help to explain why the combination of genistein and the VD3 analog 27-hydroxy-BCI-210 acts synergistically to reduce MCF-7 cells proliferation [119].

Since combination therapy is more efficacious than single treatment, the antineoplastic effect of calcitriol also has been studied with other antineoplastic compounds, such as vitamins, including vitamin A metabolites and vitamin K, whose action is described below.

### 3.5. Synergistic Antitumoral Effects of Retinoids and Calcitriol in BC

The most active metabolite of vitamin A in the family of retinoids is all-trans-retinoic acid (ATRA), commonly referred as retinoic acid, tretinoin, and vitamin A acid [120]. ATRA helps cells grow and develop, especially in the embryo, controlling the segmentation in developing organisms and the homeostasis of various tissues in the adult [121]. The biological activity of retinoids is primarily mediated by members of the nuclear retinoid acid receptors (RARs) that form heterodimers with members of the RXR, acting as ligand-activated transcription factors that translocate to the nucleus and bind to Retinoic-Acid-Response-Elements (RAREs) in the promoter of target genes, regulating the transcription of genes involved in cell growth and differentiation [122]. Based on the above, the deregulation of retinoids signaling pathways, including the malfunction of RARs, have been closely related to tumorigenesis, while retinoid administration is related to the inhibition or reversion of carcinogenic process in hematological cancers, premalignant lesions in the oral cavity, head and neck squamous cell carcinoma, neuroblastoma, ovarian, bladder, liver, skin, prostate, and BC [120,121,122,123,124]. ATRA exerts anti-inflammatory, antiangiogenic, and anticancer effects. The anticancer effects of retinoids include inhibition of proliferation, induction of apoptosis, and differentiation of cancer cells [120]. ATRA was first clinically useful as a differentiation agent to treat acute promyelocytic leukemia [125]; however, its therapeutic use is limited, since it may generate systemic toxicity, induce teratogenicity or chemical hepatitis. These unwanted side effects could be avoided by agents capable of preserving/increasing its antitumoral effects while allowing to reduce its dose. In this regard, the antitumoral effect of ATRA has been evaluated in combination with calcitriol in BC cells, showing a synergistically growth inhibitory effect upon T-47D [126], and MCF-7 cells [82]. Moreover, the combination of ATRA with calcitriol acted synergistically to inhibit the clonogenicity of MCF-7 and T-47D cell lines, both expressing ER, VDR, RAR, and RXR. Interestingly, in MDA-MB-231 TNBC cells, which lack the expression of RARα and RARβ, the combinatorial effect of ATRA and calcitriol was additive. In addition, the treatment of calcitriol and ATRA, either individually or combined, sensitized BC cells to the effects of paclitaxel and adriamycin, chemotherapeutic agents commonly used in the treatment of BC [52]. Additionally, the combination of calcitriol and ATRA induced a more differentiated phenotype in BC cells, with additive effects in a function and cell-specific manner [127].

### 3.6. Vitamin K3 (Menadione) Sensitizes BC Cells to the Growth Inhibitory Effects of Calcitriol

Vitamin K, an essential nutrient, is identified as a cofactor that participates in normal blood coagulation and bone metabolism and has exhibited potent anticancer activity. The members of the vitamin K family are phylloquinone (K1), menaquinone (K2), and menadione (K3). The latter is not properly considered a natural vitamin K, but rather a synthetic analog that cannot exert all the functions of vitamin K [128]. While all vitamin K family members exhibit antineoplastic effects, most anticancer research has focused on menadione, whose effectiveness has been studied in different neoplasms including those of the breast, prostate, bladder, liver, blood, and oral cavity [128,129,130]. Regarding BC, menadione inhibits cell growth, promotes apoptosis, and arrests the cell cycle regardless of BC molecular subtype. Menadione mechanism of action involves the generation of ROS, damaging the mitochondria, inducing apoptosis, and reducing cell survival factors. Additionally, this vitamin modifies cellular nucleophiles such as cysteine residues on proteins and promotes apoptosis through caspase-3 or poly (ADP-ribose) polymerase (PARP) cleavage, arresting the cell cycle [77,129]. The combination of menadione with different chemotherapeutic agents has been shown to elicit additive and synergistic effects. When menadione and calcitriol are combined, the antiproliferative effect in MCF-7 BC cells is enhanced compared to each drug alone, which may be caused, at least in part, by triggering oxidative stress, as suggested by increased ROS production [77]. In vivo, the combined treatment of menadione with calcitriol delayed murine TNBC tumor growth more efficiently than mono-treatments, which was associated with increased tumor cells death [131]. Similarly, the co-treatment of menadione with calcitriol has shown to increase the antiproliferative effect in MCF-7 BC cells by promoting oxidative/nitrosative stress, mitochondrial alteration, and autophagy [78].

### 3.7. Combined Use of Calcitriol and Fatty Acid in BC

Both omega-3 free fatty acids and VD3 play a positive role in the reduction of BC incidence. Based on the above, Yang and colleagues evaluated in 2017 the combinatorial effect of omega-3 free fatty acids and calcitriol. This combination suppressed cell proliferation and enhanced cell apoptosis among three subtypes of BC cell lines: ER and PR-positive, HER2-positive, and TNBC. The mechanism of action depended on caspase signals and Raf-MAPK signaling pathway activation [79]. Despite the beneficial effects of aromatase inhibitors (AIs) in the treatment and survival outcomes in BC, its use induces arthralgia, leading to drug discontinuation. Fortunately, the use of omega-3-fatty acids has been associated with significantly lower pain scores in obese patients with BC. Additionally, some studies have demonstrated that patients with insufficient or deficient levels of VD are more likely to experience arthralgia during AIs treatment [132]. Therefore, the combination of VD or its derivates with omega-3-fatty acids could be beneficial in adjuvancy withAIs.

The concluding remarks to the above combinatorial strategies are: (a) Combining calcitriol with natural compounds increase antineoplastic effects (b) the described compounds are not cytotoxic; therefore, could be used at their optimal doses to exert the therapeutic effect without developing undesirable side effect and decreasing the possibility that resistance will generate. (c) The combined treatment of calcitriol with specific natural compounds, such as curcumin, modulates drug metabolism resulting in increased anticancer activity.

## 4. Calcitriol in Combination with Endocrine Therapy

Endocrine therapy has been used for the management of early and advanced hormone-positive BC. This therapy functions by blocking the estrogen signaling or by inhibiting estrogen synthesis. Among the most commonly used endocrine therapeutic factors are tamoxifen, raloxifene; fulvestrant; and AIs [133].

The antiestrogens tamoxifen and fulvestrant competitively inhibit the binding of estradiol to the ER. Tamoxifen induces changes in ER conformation resulting in the recruitment of coactivators or corepressors. Depending on the interaction of ER-tamoxifen complex, tamoxifen can act as either partial agonists or antagonists of ER function in a tissue-, cell-, and promoter-specific manner. Due to these selective activities, tamoxifen is classified as a selective estrogen receptor modulator (SERM). Based on its antagonist action, it is used for the treatment of women with metastatic BC, as adjuvant therapy of primary BC, as well as for the reduction of BC risk [134,135,136]. Fulvestrant, on the other hand, has no agonistic effects, since it interrupts ER dimerization and nuclear localization, blocking ER-mediated transcriptional activity associated with tumor progression, invasion, metastasis, and angiogenesis. This antiestrogen also accelerates receptor degradation, and therefore is considered as a selective estrogen receptor down-regulator (SERD) [137]. Fulvestrant is used to treat hormone receptor-positive advanced BC in postmenopausal women without previous endocrine therapy or with disease progression following endocrine therapy [138].

In vitro studies in MCF-7 and ZR-75-1 BC cell lines have shown that the combined treatment of calcitriol and tamoxifen inhibited, in a cytostatic way, cell proliferation to a greater extent than either compound alone [139], through inducing apoptosis [140]. This combination allowed reducing the doses of calcitriol. Moreover, calcitriol diminished the estradiol-stimulated growth of the two ER-positive cell lines. The pharmacological effect of both compounds was classified as an additive interaction [139,140,141]. Interestingly, tamoxifen treatment increased in a dose-dependent fashion the levels of VDR, thus favoring calcitriol biological effects [142].

Notably, theVD3 analog EB1089 is more potent to inhibit the proliferation of MCF-7 cells compared to calcitriol. In MCF-7 cells stimulated with estradiol, the co-treatment of EB1089 with fulvestrant suppressed the estradiol-stimulated growth of MCF-7 cells and produced a higher inhibitory effect than either compound alone [143]. 22-oxa-l,25-dihydroxyvitamin D3, (22-oxa-calcitriol) is another synthetic analog of calcitriol that inhibits BC cell growth regardless of ER status without raising serum calcium concentrations [144,145]. Combining this analog with tamoxifen enhanced the 22-oxa-calcitriol antitumor effect in an ER-positive BC model [145]. In addition, Ro24-553, another calcitriol analog, inhibits mammary carcinogenesis by extending tumor latency and reducing tumor incidence. Its combination with tamoxifen in a murine model increased the anti-estrogenic actions of tamoxifen, resulting in the reduction of tumor burden and incidence [146].

On the other hand, within the pathophysiology of BC, it is known that it can metastasize to the bones. A risk factor related to this is the increase of bone resorption [147]. In this sense, it was demonstrated that calcitriol and its analogs, EB1089 and KH1060, stimulated calcium release in a dose-dependent manner from long bones of fetal mice. Significantly, tamoxifen, or fulvestrant treatment inhibited the bone resorption promoted by calcitriol and its analogs [141,148]. Hence, the potential side effect of treating BC patients with calcitriol or its analogs is the increased risk of skeletal metastases due to the stimulation of bone resorption, which could be reduced by combining them with antiestrogens, thus taking advantage of the antiproliferative proprieties of VD compounds.

An important antitumoral mechanism of tamoxifen is the reduction of glucose uptake. However, calcitriol co-treatment was found to significantly attenuate this effect in MCF-7 cells. In order to avoid this, combining calcitriol with Glucose-6-phosohate dehydrogenase-inhibiting regimens could improve substantial antitumor effects observed by the combination [55].

### 4.1. Calcitriol or Its Analogs in Combination with AIs

The AIs anastrozole, letrozole, and exemestane are prescribed to postmenopausal women with hormone receptor-positive BC. They inhibit the expression of aromatase, the enzyme that catalyzes the conversion of androgens to estrogens, thereby decreasing circulating estrogens’ levels [149].

Several studies have demonstrated that calcitriol or EB1089 can also suppress aromatase expression and activity, resulting in the reduction of estrogen synthesis in different ER-positive BC cell lines (MCF-7, ZR-75-1, and T-47D) [150,151,152,153,154]. The combination of calcitriol or its analog EB1089 with exemestane, anastrozole, and letrozole has been shown to inhibit the growth of the MCF-7 cell line [152,153]. Moreover, the combined treatment of calcitriol and the AIs also reduced the tumor growth of MCF-7 xenografts, as compared to the administration of compounds alone. The mechanism involved was the downregulation of aromatase and ER expression. The combination of calcitriol with AIsalso has anti-inflammatory and antiproliferative effects mediated by the negative modulation of the cyclooxygenase (COX)-2 and the upregulation of insulin-like growth factor binding protein 3 (*IGFBP-3*), and *p21* gene expression [154].

Another study has demonstrated that the VD active metabolite (24R)1,24-dihydroxycholecalciferol (PRI-2191) and the analog PRI-2205 significantly enhanced the antitumor activity of anastrozole in BC tumors and cells [155].

### 4.2. Calcitriol and Resistance to Endocrine Therapy

The development of resistance towards antiestrogens may occur de novo or may be acquired during the treatment, representing a significant clinical problem. The mechanisms implicated in endocrine resistance include regulation of signal transduction pathways, the balance of co-regulatory proteins, loss or modification in ERα expression, altered expression of specific microRNAs, and genetic polymorphisms involved in tamoxifen metabolic activity [156].

Interestingly, in BC cell lines resistant to antiestrogens, calcitriol and EB1089 inhibit cell proliferation by inducing growth arrest and apoptosis [157,158]. In fact, EB1089 was more potent to inhibit cell proliferation in antiestrogens-resistant cell lines than in parent cells [157,159]. These studies indicated that the sensitivity to VD analogs might increase after developing antiestrogen resistance and vice versa [159]. Another study demonstrated that calcitriol decreased the cell growth of tamoxifen-sensitive and -resistant BC cells by inhibiting the NFκβ pathway through increased gene expression of NFκβ inhibiting protein IκB, with a concomitant reduction of tumor necrosis factor alpha (TNFα)-induced p65 phosphorylation, as well as its translocation into the nucleus [160].

In addition to the downregulation of aromatase by calcitriol and EB1089 [150,151,152,153,154], these compounds can decrease ER expression in BC cells, attenuating the estrogen signaling [55,143,154,161,162,163,164]. Consequently, these antineoplastic proprieties can improve the antiproliferative inhibitory response of endocrine therapy in ER-positive BC. Interestingly, in cultured ER-negative breast tumor-derived cells and in an endocrine therapy-non-responsive BC cell line, previous work from our laboratory demonstrated that calcitriol pre-treatment restored the ability of antiestrogens to inhibit cell proliferation in the ER-negative BC cells, through inducing ERα expression. Moreover, calcitriol combined with fulvestrant downregulated *ether-a-go-go*-1 potassium channel (*EAG1*) and *CCND1* gene expression; both molecules are important in cell cycle regulation and tumor progression [165]. The mechanism involved in the calcitriol-dependent ERα induction in ER-negative BC cells implicates the direct interaction of the VDR-RXR complex to VDREs in the ERα gene promoter region, including the inhibition of histone deacetylases (HDAC) and DNA methyltransferase (DNMT) enzymatic activity [166].

As a summary of this section, calcitriol and endocrine therapies in combination provide several potential advantages, such as increasing growth arrest, inducing apoptosis, and evoking anti-inflammatory and antiproliferative effects. In addition, the ability of calcitriol to induce ER expression in ER-negative tumor cells plays a primordial role in the re-sensitization to endocrine therapies, and implies the reduction of adverse events such as bone loss, genitourinary atrophy and musculoskeletal symptoms.

## 5. Calcitriol in Combination with Histone Modifiers

Histone modification can be defined as a post-translational alterations at the N-terminal histone tails; acetylation and methylation are the two most recognized mechanisms regulating the epigenetic effects of gene expression, genomic stability, DNA damage response, and cell cycle checkpoint integrity. Both mechanisms are importantly related to cancer development [167].

HDACs are enzymes associated with transcriptional repression. Histone deacetylase inhibitors (HDACI) are a class of compounds that interfere with the function of HDAC, inducing a hyperacetylation status of chromatin; the above disturbs the gene expression through modulating the chromatin structure [168]. It has been widely reported that changes in cancer cells lead to hypomethylation and hypermethylation of specific DNA regions, mainly within the promoters of tumor suppressor genes [169]. Trichostatin A (TSA or 7-(4-(dimethylamino)phenyl)-N-hydroxy-4,6-dimethyl-7-oxohepta-2,4-dienamide) is the most potent HDACI chemical agent that has been discovered and widely employed in BC models [170]. In different BC cell lines, this compound increases CYP24A1 expression in concentrations between 3 to 400 nM [171]. The latter points out that the use of HDACI can perturb the effects of VD-derived compounds by decreasing its bioavailability, and with this its antineoplastic effects.

## 6. Calcitriol in Combination with Kinase Inhibitors in BC

Different types of kinases are implicated in the growth of BC cells. MAPK, PI3K/AKT signaling pathway, Janus kinase (JAK)–signal transducer, and activator of transcription (STAT) pathway are mitogenic routes that have outstanding participation in cancer cell proliferation. These and other cellular signaling pathways are stimulated after the activation of various growth receptors [172,173]. Specifically, in HER2-positive and TNBC cells, the overexpression and hyperactivation of different epidermal growth factor receptor family members such as EGFR and HER2 are common. The co-expression of these receptors confers poor outcomes and a high rate of metastasis. It is important to mention that these receptors are activated by a series of phosphorylations in their tyrosine kinase residues. Thus, drugs known as TKIs are generally employed to counter their activation. It has been reported that the combination of calcitriol or different analogs with tyrosine kinase inhibitors such as gefitinib, lapatinib, or neratinib resulted in a greater antiproliferative and apoptotic effect than either drug alone in TNBC and HER2-positive BC cells [20,174]. Notably, the combination of calcitriol with different TKIs downregulated the MAPK and PI3K phosphorylation [175]. The overall synergistic effect of the combined treatment of calcitriol with TKIs can be attributed to the presence of VDREs in EGFR promoter, which regulate the expression of growth factor receptors. Moreover, calcitriol can avoid the binding of different ligands to EGFR [175,176]. Regarding this point, it has been described that calcitriol can modulate the activation of MAPK by non-genomic routes [6,177]. The coadministration of calcitriol with gefitinib and with gefitinib plus dexamethasone has also been probed in clinical trials involving solid tumors such as BC [178,179]. However, these studies were focused on evaluating the maximum tolerated dose (MTD) of calcitriol in a combined scheme administration. The authors reported no antitumor activity in patients with solid tumors when the drugs were administered together.

In combination with dovitinib, a multi-kinase inhibitor, calcitriol has also been demonstrated to have a synergistic antiproliferative effect in TNBC in in vitro and in vivo models. At the molecular level, the combination of these compounds induced cell death and inhibited tumor growth of BC cells to a greater extent than each compound alone [22]. Of note, at clinically achievable and safe concentrations, the combination of calcitriol with dovitinib allowed reducing the dose of the kinase inhibitor while preserving its antiproliferative effect. The latter suggested that lower dovitinib dosing is feasible by the co-treatment, which may decrease its adverse effects and avoid the generation of resistance in therapeutic applications.

On the other hand, the synergistic effect on cell proliferation of calcitriol in combination with ruxolitinib, a JAK1 and JAK2 inhibitor, was also demonstrated in BC cells with or without the presence of ER [180]. The combined treatment negatively modulated the protein levels of JAK2, phosphorylated JAK2, c-Myc protein, CCND1m and induced the apoptosis regulator Bcl-2, Bcl-2-like protein 1, and caspase-3 [181]. These findings indicate that the combination of TKIs with calcitriol can be favored in ER-negative BC cells, while its combination with other kinds of inhibitors such as ruxolitinib can be helpful to both panoramas. In fact, the combination of calcitriol with kinase inhibitors has shown promising results in different types of cancer [181].

In conclusion, the simultaneous treatment of calcitriol or its analogs with TKI’s in TNBC and HER2-positive BC cells is significantly better than monotherapy as antineoplastic treatment, resulting in a greater antiproliferative and pro-apoptotic effect. Part of the increased effect could be attributed to the regulation of growth factor receptors expression and activation by calcitriol. The overall preclinical evidence provides the basis for the potential use of this therapeutic combination in BC patients whose tumors overexpress TK receptors.

## 7. Calcitriol in Combination with Non-Steroidal Analgesic Drugs in BC

The sustained inflammatory environment in the cancer context is associated with enhanced cell proliferation and carcinogenesis promotion. In fact, the employment of different anti-inflammatory molecules, including nonsteroidal anti-inflammatory drugs (NSAIDs), for counteracting the inflammatory status has contributed to reducing the risk and incidence of several cancers and to inhibit cancer growth [182,183]. The NSAIDs inhibit COX enzyme activity, which exists as two isoforms: COX-1 and COX-2. The first is expressed ubiquitously in many tissues and cell types, while the second one is induced by a variety of stimuli and is involved in inflammatory processes. COX-1 and COX-2 convert arachidonic acid to prostaglandins, which promote proliferation, inflammation and play an essential role in neoplasms development and progression, including BC [184,185,186]. On the other hand, calcitriol exhibits significant anti-inflammatory actions that contribute to its antineoplastic effects [187]. For many years, different kinds of pro-inflammatory molecules such as prostaglandins and thromboxanes have been associated with bad prognosis, recurrence of the disease, and poor survival rate in patients with BC [188,189,190]. Different studies have reported that the combination of calcitriol with celecoxib, an inhibitor of COX-2, significantly reduced BC cell proliferation in a synergistic manner as compared to each single agent; an effect that was independent of ER presence [81,191]. In addition, calcitriol can downregulate COX-2 protein and gene expression in BC cell lines with or without ER expression [81,191], an effect that was attributed to its immunomodulatory role and the link between VD3 and prostaglandin metabolism [192,193].

Related to the above, the antiangiogenic effects of calcitriol may be mediated by the inhibition of prostaglandins, which are important proangiogenic factors [187], in addition to the modulation of vascular endothelial growth factor (VEGF) [194]. 

In conclusion and considering that inflammation is considered one of the hallmarks of cancer, the anti-inflammatory compounds have been widely evaluated as antineoplastic agents alone or combined with calcitriol, resulting in synergistic antiproliferative effects independently of the BC phenotype. Therefore, further studies are necessary to determine the benefit of this therapeutic strategy.

## 8. Calcitriol in Combination with Immunomodulatory Agents in BC

There are few reports on the combination of calcitriol with immunomodulatory agents in BC. However, in order to counteract the hypercalcemic effect evoked by calcitriol, schemes based on glucocorticoids have emerged regarding this point [195,196]. In addition, glucocorticoids enhance VDR transcription in many cell types [197,198]. Thus, different combinations of calcitriol with these agents have been explored, specifically in prostate cancer. The results are controversial, some of them pointing out that the combination of dexamethasone is safe, feasible, and has antitumor activity [195], while others report a lack of significant antitumoral activity in prostate cancer [199]. However, in BC cells, pre-clinical studies demonstrated that the combination of dexamethasone synergizes the antitumoral effects of calcitriol [82].

On the other hand, the role of calcitriol and its receptor has shown crucial activity in the proper activation of the immune system, particularly for T-cell development, differentiation, polarization, and function [200,201]. In BC, tumor-infiltrating lymphocytes (TIL) play an important role against cancer cells in the tumor microenvironment; however, depending on the cellular signals, TILs can modify their phenotype and exhibit pro-tumoral actions [202]. In this regard, in an orthotopic BC mouse model, it has been demonstrated that VD3 supplementation accompanied with a low-fat diet can avoid the progression of BC tumors. In contrast, in a regimen based on a high-fat diet also combined with VD3 supplementation, the growth of mammary tumors was evident. The above was correlated with changes in the activation status and infiltration of T CD8+ lymphocytes promoted by the inflammatory conditions associated with overweight. Importantly, VD supplementation also showed a reduction of both adipogenic markers and pro-inflammatory cytokines [203]. These findings add different mechanisms of action of how VD3 supplementation can slow down the growth and development of tumors of mammary origin.

In addition, a vast number of immune cells express the CYP27B1 enzyme and the VDR, which favor both the conversion of circulating calcidiol into the active form and its intracellular signaling, respectively [204,205,206]. In relation to this, it is important to remark that VD3 deficiency has correlated with a lack of successful response to immune checkpoint inhibitors (anti-PD-1, anti-PD-L1, or anti-CTLA4) in patients with metastatic renal carcinoma as compared with patients with high VD3 serum levels [207]. The above prompts to consider that VD3 supplementation is an important adjuvant strategy to avoid the prevalence of cancer. In addition, VD3 supplementation could favor the therapeutic response in the onco-immuno-biological background. Of note, the relationship between hypovitaminosis of VD3 with immunotherapy in the BC context has been scarcely explored.

It has been demonstrated that CB1093 analog improves the responsiveness of BC cells to TNFα-induced cell death by promoting TNFα-induced cytosolic phospholipase A2 (PLA2) activation [208]. Similarly, results from our laboratory demonstrated that the combination of calcitriol with TNFα resulted in a more significant antiproliferative effect than the drug alone in ER-positive and ER-negative BC cells [209].

Considering the magnitude of the problems generated by VD deficiency, and taking into account all the benefits of calcitriol as an antineoplastic and immunomodulatory agent, it is highly recommended to assess VD serum levels followed by its supplementation when necessary, in women with high risk of BC development or in-treatment for this pathology.

## 9. Calcitriol in Combination with Histamine Inhibitors in BC

Histamine is one of the first proinflammatory mediators to be described, and its primary sources are basophils and mast cells, which are distributed widely in the skin and mucosa. In response to allergic stimuli, a complex interaction between inflammatory cells is activated, and several inflammatory mediators are produced; among these, histamine, which regulates the maturation and activation of leukocytes and directs their migration to target sites where they cause chronic inflammation [210]. Additionally, in vivo and in vitro studies have described that histamine is involved in cell proliferation, migration, and invasion of several cancers [211]. Accordingly, the use of antihistamines has shown promising effects to fight this pathology, as in the case of astemizole, a non-sedating second-generation antihistamine, commonly prescribed for the treatment of allergies. This drug has been repurposed as an antineoplastic agent, since in addition to H1-histamine receptors blockade, it targets several other molecules involved in cancer development, such as P-glycoprotein and the voltage-gated potassium channels EAG1 and human EAG related genes (HERG) [212]. Regarding BC, astemizole has shown to exert antiproliferative effects against both hormone-dependent and non-hormone-dependent BC cell lines, as well as in primary cell cultures derived from breast tumors [21,83]. The antineoplastic effects of astemizole also have been evaluated in conjoint with other antineoplastic agents, including calcitriol [21,83,213]. In BC cells, this antihistamine compound synergized the antiproliferative activity of calcitriol by downregulating CYP24A1, upregulating the VDR, and targeting *EAG1* [83]. Moreover, in vivo studies showed that the co-administration of astemizole and calcitriol to mice xenografted with human BC cells inhibited tumor growth more efficiently than each drug alone [213]. In summary, the therapeutic use of this antihistamine with calcitriol could be beneficial as adjuvant therapy for BC, independently of the tumor phenotype, since the molecular targets of these compounds are the VDR and EAG1 channel, both of them highly expressed in BC.

**Figure 1:**
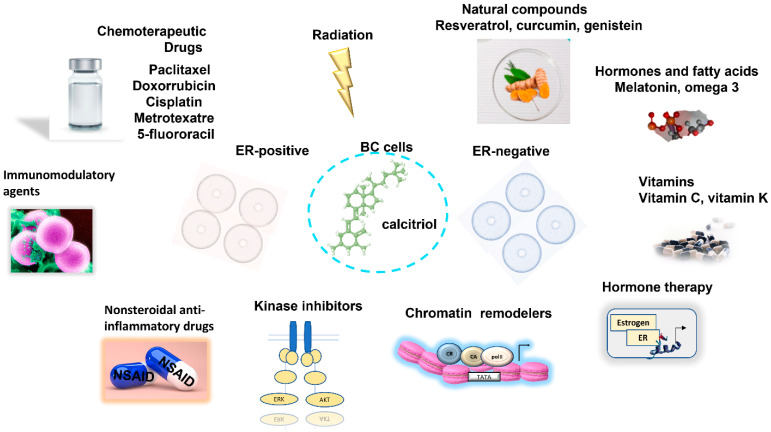
Combination schemes of calcitriol with different anti-cancer treatments. The combination of calcitriol with different chemotherapeutic drugs, radiation, hormones, vitamins, chromatin remodelers, target therapy, nonsteroidal anti-inflammatory drugs, or immune therapy has been evaluated in hormone and non-hormone dependent breast cancer models. Most of these combinations have reported synergistic effects to decrease cancer cell proliferation, induce apoptosis, avoid angiogenesis and invasion, increase radiosensitivity, inhibit the stem cell phenotype, change the cell metabolism and inhibit mitogenic pathways.

In Figure 1 below, we provide a scheme of the different combination regimens of calcitriol with the agents mentioned in this review in BC.

## 10. In Vivo Preclinical and Clinical Studies with Calcitriol-Based Regimens in BC

Calcitriol has shown significant antitumor activity in preclinical BC research in animal models. Furthermore, many antineoplastic mechanisms previously reported in vitro have been corroborated at the preclinical level in vivo. The animal models have allowed establishing the concept that the administration of calcitriol in intermittent doses is fundamental to avoid its unwanted calcemic effects. In fact, different schemes of doses of calcitriol have also been tested in rat and murine mammary carcinogenesis models. Additionally, in vivo experiments have also pointed out the importance of developing different calcitriol analogs able to maintain its antiproliferative activities without inducing hypercalcemia (Table 1).

Since the 90s, the effect of calcitriol in BC models has been investigated. For instance, a rat mammary cancer model induced by N-methylnitrosourea (NMU) was used by Colston et al. in 1992 to study the antitumor effect of calcitriol or its synthetic analogs alfacalcidol and calcipotriol in BC-tumor bearing rats, showing a decrease in tumor growth in all cases. The development of hypercalcemia was reported in the case of calcitriol and alfacalcidol at the doses tested, but only a slight increase was observed when using calcipotriol [214]. Accordingly, different studies employing xenograft models with ER-positive and ER-negative BC cells have reported that the supplementation with VD3 as well as the administration of calcitriol or different analogs exert antitumor effects [37,38,74,154,215,216].

In the case of ER-positive BC xenografts, the antitumor effects of calcitriol have been evaluated alone or in combination with AIs such as anastrozole and letrozole. Calcitriol alone demonstrated a great reduction in the tumor growth, although its combination with anastrozole and letrozole caused a statistically significant tumor inhibition compared to the single agents. Interestingly, calcitriol decreased the aromatase expression and estrogen levels in xenograft tumors and mammary adipose tissue, reflecting its ability to disrupt estrogen survival signals. Different doses of calcitriol were administered intraperitoneally in an intermittent scheme (0.025, 0.05, and 0.1 µg doses three times a week), while the AIs were administered six days a week, for four weeks. The growth was greatly reduced in the combined scheme as compared with calcitriol alone [154].

On the other hand, the antitumoral action of VD3 has also been evaluated in different models. In this regard, the ingestion of a VD3-supplemented diet (5000 IU/kg) compared with a control diet (1000 IU/kg) was tested on immunocompromised mice bearing ER-positive BC xenografts. This regimen of dietary VD3 intake was also compared with different doses of calcitriol, including 0.025, 0.05, or 0.1 μg/mouse, three times a week. Both treatments displayed similar effects in the inhibition of tumor growth in mice. In addition, both calcitriol and dietary VD3 were equipotent in suppressing estrogen synthesis (inhibiting aromatase expression) and signaling (reduction of serum levels of estradiol). In addition, the VD3-compounds also reduced proinflammatory factors and growth signaling pathways such as COX-2, 15-hydroxyprostaglandin dehydrogenase (15-PGDH), prostaglandin E receptor (EP), prostaglandin F receptor (FP), p21 among other proteins [37], which suggested that VD3 or calcitriol administration may have a beneficial antitumor effect. Of note, as previously discussed, different analogs of calcitriol have been demonstrated to elicit antitumor action in mice bearing human ER-positive BC cells. Nevertheless, these effects were not reproducible in ER-positive mammary tumor cells of murine origin, such as the 4T1 cell line. Regarding this point, two analogs of calcitriol, PRI-2191, and PRI-2205 were administered in BALB/c female mice orthotopically inoculated with 4T1 cells. The analogs of calcitriol were administered subcutaneously thrice a week starting from day 7 after tumor cell inoculation. The single dose of compounds was as follows: calcitriol, 0.5 µg/kg; PRI-2191, 1.0 µg/kg; and PRI-2205, 10.0 µg/kg. Of note, no evident antitumoral effect was reported in this study [217], pointing out that mammary characteristics between murine and human tumors are different and deserve to be carefully considered.

Regarding the effects of calcitriol in TNBC in vivo models, different articles have also demonstrated that calcitriol or its analogs can inhibit tumor growth as in xenograft models where ER-positive BC cells have been used [216]. The antitumoral actions of calcitriol or different analogs in TNBC cell lines have been associated with the elevation of protein levels of cyclin-dependent kinase inhibitors including p27 and p21 as well as induction of apoptosis mediated by PARP cleavage.

Moreover, the use of different analogs of calcitriol has emerged in recent years to avoid its possible calcemic effects. EB1089 was demonstrated to inhibit the tumor growth of estrogen-independent invasive cells and tumors, retaining the antiproliferative and proapoptotic effects of calcitriol. EB1089 was tested in two ways, implanted pellets, and subcutaneous injection for four weeks. Both routes of administration showed to reduce tumor growth; however, the data suggested that pellet delivery may minimize the calcemic side effects of VD3, as mice treated with this form presented lower calcium serum levels compared with the subcutaneous injection [216]. Another analog of calcitriol that has also probed antitumoral actions in TNBC xenograft models is Gemini 0097. This compound demonstrated to reduce tumor growth by 60% without causing hypercalcemia [38]. The dose of Gemini 0097 administered to the immunocompromised mice was 0.1 μg/kg body weight in 0.1 mL vehicle, which was daily injected intraperitoneally from day 4 until the termination of the experiment. Gemini 0097 upregulated the protein expression of an inhibitor of cell cycle p21 and IGFBP-3 [38]. Thus, analogs of calcitriol may represent useful alternatives, either alone or in combination with other therapeutic agents, for treating ER-positive BC.

Combinations of calcitriol with other agents such as curcumin and resveratrol have been performed in a TNBC cell xenograft model [74]. In this study, calcitriol was intraperitoneally administrated 0.25 µg in 100 µL once a week. Curcumin was administered daily in the drinking water at 40 mg/kg (throughout the experiment), and resveratrol was orally administered 1.2 g/kg three times a week. All treatments alone or in combination were given for three weeks. The general results showed decreased tumor onset, volume, and micro-vessel density in mice co-administered with calcitriol and either curcumin or resveratrol [74]. This work highlighted the importance of spaced calcitriol administration generating a good antitumor response without causing calcemic effects. In addition, calcitriol has also been recently evaluated in combination with dovitinib in a human-derived TNBC xenograft mice model. Calcitriol was also intraperitoneally administered 0.25 μg/100 μL every week, while dovitinib was intraperitoneally administered at 20 mg/kg twice per week. The treatment was followed for three weeks. Again, the results indicated that this administration scheme allowed antitumoral effects without hypercalcemia in the experimental animal groups that received calcitriol alone or combined with dovitinib [22].

On the preclinical studies, different schemes of calcitriol administration and combinations with several therapeutic drugs have been tested to reduce its side effects while trying to achieve the antitumor effects of this hormone. In general, the conclusions are that the antineoplastic activity of calcitriol is dose-dependent and, in most systems, concentrations of 1 nM or higher are associated with significant antineoplastic activity in vitro. Many reports support that the daily oral administration of calcitriol is not recommended to achieve similar effective concentrations as found in vitro due to calcemic effects [218]. Thus, different intermittent doses and the route of administration of calcitriol are affordable ways to achieve peak blood concentrations of calcitriol of approximately 0.7 nM, similar to that reported in in vitro studies. Supporting this fact, Smith et al. evaluated the subcutaneous calcitriol administration every other day in doses ranging from 2 to 10 μg for four months in patients with advanced malignancies. They assessed the pharmacokinetics of calcitriol on days 1 and 7 in the first week, and other blood parameters were weekly monitored until the end of the study. The authors reported that hypercalciuria was a common side effect found in most enrolled participants. Moreover, when the patients received 10 μg calcitriol, all of them presented hypercalcemia. The authors indicated that substantial doses of calcitriol could be administered subcutaneously with tolerable toxicity [218]. In addition to the above, Beer and colleagues reported the feasibility of dose escalation of calcitriol in patients with refractory malignancies. The aim of their work was to determine the range of escalation doses of calcitriol administrated orally to cancer patients and establish an ideal dose of it for future evaluations. According to this, the patients received four weeks of oral, weekly calcitriol treatment from 0.06–2.8 μg/kg. The authors concluded that the dose of 0.5 μg/kg was selected for future evaluations in Phase II studies, avoiding the side effects of calcitriol [219].

Escalation dose of calcitriol has also been evaluated in combination with paclitaxel in patients with advanced solid tumors. Muindi et al. considered low (4, 6, 8 μg/kg), medium (11, 13, 17, 22 μg/kg), and high (29 and 38 μg/kg) profiles of doses of calcitriol for escalation. Patients received oral calcitriol on days 1, 2, and 3 every week, while paclitaxel (80 mg/m^2^) was intravenously infused on day 1 in the first week or day 3 for the following weeks. The treatment was divided into cycles consisting of 6 weeks followed by two-week period without treatment; nevertheless, the total duration of the study was not mentioned. The authors reported that there was no dose-limiting toxicity in the trial. Even the higher dose of calcitriol (38 μg), which was administered for three days each week, did not show clinically significant hypercalcemia [220]. The authors concluded that very high doses of calcitriol can be safely administered in combination with paclitaxel, and that the achieved high serum calcitriol levels approached those previously reported to potentiate taxanes and platinum analogs cytotoxicity. Nevertheless, it is noteworthy to mention that the authors pointed out an outstanding interpatient variability in serum concentrations of calcitriol after oral administration. However, serum concentrations of this hormone were maintained 24 h after its administration. Some limitations of this study can be highlighted. First, the patients needed to swallow around 22–76 capsules of calcitriol to achieve serum concentrations of 11 to 38 μg of calcitriol, which surely resulted in a lack of adherence to treatment. Second, the study was stopped; thus, the MTD of calcitriol was not determined, and no dose-limiting toxicity has been encountered under this escalation scheme [220]. The authors did not precisely discuss the antitumor effect of calcitriol in patients with advanced solid tumors.

Complementing the previous study, the research group of Muindi et al. in 2005 investigated the pharmacokinetics of a liquid formulation of calcitriol in patients with advanced solid tumors compared to a caplet formulation that they previously evaluated in 2002 [221]. They employed a weekly intermittent schedule based on calcitriol administration 1–3 days a week (QDx3). In conclusion, the authors demonstrated that the clinical use of liquid formulation to deliver high doses of calcitriol is associated with diarrhea and does not offer greater advantages in pharmacokinetic or bioavailability terms over the use of the caplet formulation. Additionally, they confirmed that hypercalcemia was not the dose-limiting toxicity on a QDx3 weekly intermittent treatment of calcitriol schedule [221].

On the other hand, the research group of Beer et al. evaluated a different oral high dose formulation of calcitriol (DN-101, which contains 15 or 45 μg per capsule) in patients with advanced cancer. Different cohorts of patients received doses of 15, 30, 45, 60, 75, 90, 105, 135, 165, 210, 270 and 345 μg of calcitriol. In this report, the dose of 45 μg was established as the MTD. With this regimen, there were no patients with hypercalcemia as limiting toxicity, whereas in patients treated with 60 μg of calcitriol, hypercalcemia was reported as a common side effect. Thus, the authors assumed that weekly doses of DN101 at 45 μg were well tolerated in patients with cancer [222].

Specifically, in patients with BC, calcitriol administration independently or in combination has been scarcely studied. Observational studies, systematic reviews, and meta-analyses have established a strong inverse association between circulating calcidiol and BC risk [223,224]. In fact, data derived from these studies suggested that calcidiol serum levels around 52 ng/mL or more were associated with a 50% reduction in BC risk [225,226]. However, many other works have reported non-conclusive associations, with a better outcome related to changes in mammography density of patients with BC [219,220,221,222,223,224,227,228]. As we mentioned previously, different clinical studies employing calcitriol have been performed in patients with cancer, specifically in solid tumors [229]. However, few studies performed on patients with BC have focused on finding the MTD of calcitriol. As an example of the above, Fakih et al. and Muindi et al. studied calcitriol administration in combination withTKIs. They have mainly reported that the administration of gefitinib in patients with BC allowed the safe escalation of calcitriol to the MTD of 125 μg/week [178,179].

On the other hand, AIs suppress the peripheral conversion of androgen to estrogen by inhibiting the aromatase enzyme, which results in a significant estrogen decrease. Accordingly, accelerated bone loss due to the absence of estrogen leads to a lower bone density and increased fracture risk. On the other hand, and as mentioned earlier, calcitriol has been shown to inhibit aromatase expression in vitro and in vivo [154]. Thus, the combination of AIsand calcitriol has resulted in a better tumor growth inhibition at the preclinical level [151]. Bisphosphonates are drugs usually employed in the prevention of loss of bone mass. This was the rationale of a study that evaluated the combination of bisphosphonate, alendronate, and calcitriol in postmenopausal women with early BC receiving AIs [230], since these compounds commonly cause a loss of bone mass and density [231]. The results demonstrated that the combination of 5 mg of alendronate and 0.5 μg of calcitriol effectively prevented bone loss due to the aromatase inhibitor regimen in postmenopausal women with early BC. Of note, this study did not evaluate the antitumoral effect of the combination.

In postmenopausal women, treatment with tamoxifen has an estrogenic effect on maintaining bone mineral density in the lumbar spine and femoral neck with reduced fracture risk [232,233,234]. In contrast, in pre-menopausal women, bone loss is increased during tamoxifen treatment [235]. Similarly, a longer duration of aromatase inhibitor use has been associated with increased odds of developing cardiovascular disease, accelerated bone loss, and bone fractures [236,237]. Through disease-progression modeling, the analysis of the change of bone mineral density in postmenopausal patients with early BC who received postoperative hormonal therapy found that VD3 supplementation had a protective effect on osteoporosis [238].

It has been reported that the administration of 5 mg alendronate, used to treat and prevent osteoporosis, with 0.5 μg calcitriol can prevent bone loss due to AIsin postmenopausal women with early ER-positive BC [230]. Similarly, Hadji et al. recommend that women with BC under aromatase inhibitor therapy should receive calcium and VD3 supplements [239].

Interestingly, VD3 may contribute to the modification of plasma concentrations of different antineoplastics. In this regard, it has been demonstrated that calcidiol, used as a measure of VD3 status, upregulated the expression of the CYP3A4 drug-metabolizing enzyme, which in turn reduced serum levels of CYP3A4-metabolized drugs, such as letrozole [240]. However, there was no association between serum calcidiol levels, body mass index, or related markers (insulin, C-reactive protein, and leptin) and estrogen levels in patients who received standard-dose letrozole therapy [241]. In the patients who received adjuvant tamoxifen therapy, it was demonstrated that plasma levels of the tamoxifen metabolites, endoxifen, and 4-hydroxytamoxifen were reduced during winter months than across seasons [242,243], which has been associated with lower VD3 levels [242]. While in another study, no correlation was found between calcidiol plasma levels and CYP3A4 activity [243]. However, patients who received tamoxifen therapy had significantly increased serum calcidiol levels [244].

AIs, by suppressing estrogens, can cause a loss in bone mineral density and increase the risk of fractures [149,245]. They also exacerbate musculoskeletal symptoms, increasing the incidences of arthralgia and myalgia [246]. Considering the musculoskeletal adverse effects induced byAIs, the use of high doses of VD3 supplementation (50,000 IU VD3 per week) has been investigated in patients receiving letrozole. VD3 supplementation was safe and resulted in clinically significant improvement, reducing the arthralgia derived from the aromatase inhibitor pharmacological scheme [246,247]. Moreover, Altundag et al. have suggested that the combined use of VD3 and omega-3 fatty acids is a good option for reducing AIsinduced arthralgia in patients with BC [248]. Moreover, women who received adjuvant AIsand premenopausal women treated with tamoxifen with accelerated bone loss and increased fracture risk were recommended to perform weight-bearing exercise and VD3 and calcium supplementation [249]. 

Vaginal atrophy is one of the adverse events in BC women receiving tamoxifen therapy. A clinical trial in women with BC with tamoxifen-induced vaginal atrophy, demonstrated that the use of VD3 and vitamin E vaginal suppositories increased the vaginal maturation index, reduced vaginal pH, and improved symptoms of genitourinary atrophy compared with the placebo group. These data indicated that local VD3 and vitamin E improved vaginal atrophy in women with BC receiving tamoxifen [250].

It should be noted that few studies have evaluated the antitumor effect of calcitriol supplementation in postmenopausal patients with BC. In this regard, Urata et al. used samples from patients with BC before and after a short-term oral calcitriol supplementation (0.50 μg/day for 30 days) to study the expression of Ki67 protein, which is an important marker of cell proliferation. They found that Ki67 expression was reduced in 10/32 post-calcitriol samples. The authors concluded that even if calcitriol was able to modulate the expression of targets genes in some samples, it was neither sufficient to elicit an adequate antiproliferative response nor to induce the hormone transcriptional signaling pathway in BC specimens [227]. The results of this study highlighted that calcitriol supplementation deserves better attention, due to a high rate of VD3 deficiency found in the enrolled patients. We consider that the latter study has some limitations, the first is that calcitriol supplementation was performed for a short time. A second one would be that the authors did not employ an intermittent scheme of supplementation with higher doses of calcitriol, which as previously discussed, has been associated with better results. Finally, the study considered only few patients and did not consider different parameters involved in the catabolism of calcitriol, such as polymorphism of VD3 metabolic enzymes.

As summary, in Table 1 we include preclinical studies performed in animals as well as clinical studies, undertaken with calcitriol and/or its analogs, alone and in combination with different agents in BC.

**Table 1:** In vivo preclinical and clinical studies with calcitriol and/or its analogs, alone and in combination with different agents in BC.

Preclinical In Vivo Models					
Drug	Model	Doses	Aim	Results	Ref.
Calcitriol/calcipotriol/alfacalcidol	Rat mammary cancer model induced by N-Methyl-nitrosourea	Intraperitoneal administration of 0.25 μg/kg and 1.25 μg/kg thrice weekly for 28 days of calcitriol, and administration of calcipotriol (50 μg/kg) in the same time	To evaluate the effects on calcium metabolism and mammary tumor growth in adult female rats, and compare the antitumoral effects of calcitriol and its analogs calcipotriol and alfacalcidol.	All VD3 metabolites inhibited tumor growth of mammary carcinoma. However, calcitriol and alfacalcidol at the doses tested provoked hypercalcemia	[214]
Calcitriol, anastrozole, and letrozole	Murine model (control and ovariectomized mice)	Anastrozole was administered at 5 µg and letrozole at 2.5 µg six days a week. Calcitriol was administered at 0.025, 0.05, and 0.1 µg doses three times a week. All substances were given intraperitoneally for four weeks.	To investigate whether calcitriol would enhance AIs activity in vivo to inhibit the growth of MCF-7 tumor xenografts.	All three concentrations of calcitriol tested exerted significant tumor inhibitory effects, and maximal inhibition was seen with the highest dose used (0.1 µg/mouse). Of note, the combined treatments caused higher inhibition of estrogen synthesis in the tumor microenvironment as reflected by estrogen levels measured in the tumors and surrounding mammary fat. Calcitriol decreased aromatase expression in various tissues.	[154]
VD3 and calcitriol	Murine model (control and ovariectomized mice)	Oral VD3 supplemented diet (5000 IU/kg) and injections of calcitriol 0.025, 0.05, or 0.1 μg/mouse, three times a week).	To investigate the beneficial effects dietary VD3 in comparison with injections of calcitriol using xenograft models of ER-positive BC.	Both treatments displayed similar effects in the inhibition of tumor growth in mice. Both calcitriol and dietary VD3 were equipotent in suppressing estrogen synthesis and signaling, and reduction of proinflammatory factors and growth signaling pathways.	[37]
EB1089	Six-week-old ovariectomized female NCr-nu mice	EB1089 was administered in a daily subcutaneous injection (45 pmol EB1089 in propylene glycol/PBS, 4:1) or via implanted continuous release pellets delivering either 60 or 120 pmol of EB1089 per day. The total treatment lasted 4–5 weeks.	To determine the effects of calcitriol and EB1089 on the ER-negative, cell line SUM-159PT, in vitro. To determine whether EB1089 could modulate growth and/or apoptosis of ER-xenografts.	In mice implanted with EB1089 pellets, average tumor volume decreased gradually over the four weeks of treatment. The treatment with EB1089 decreased PCNA protein expression. Both forms of administration of EB1089 showed to reduce tumor growth; however, the data suggested that pellet delivery may minimize the calcemic side effects.	[216]
Calcitriol, PRI-2191, or PRI-2205	Immune-competent BALB/c female mice	The analogs of calcitriol were administered subcutaneously thrice a week starting from day 7 after tumor cell inoculation. The single dose of compounds was as follows: calcitriol, 0.5 µg/kg; PRI-2191, 1.0 µg/kg; and PRI-2205, 10.0 µg/kg.	To investigate the effect of calcitriol and its analogs on the growth and metastasis of murine mammary cancer at various progression stages (days 14, 21, 28, and 33)	Treatment with calcitriol at initial stages showed moderate lung metastasis as compared with its analogs. Nevertheless, the treatment with calcitriol or both analogs resulted in the stimulation of lung metastases. The treatments did not alter antiangiogenic and angiogenic factors thrombospondin 1 (TSP-1) and VEGF, respectively. However, they positively affected the protein expression of OPN, TGF-β, serum levels of E_2_ and diminished the expression of VDR. Calcitriol or its analogs downregulated the expression of some genes encoding for growth factors.	[217]
Calcitriol + curcumin Calcitriol + resveratrol	TNBC xenografts performed in nude female mice	Calcitriol was intraperitoneally administrated 0.25 µg in 100 µL once a week. Curcumin was administered daily in the drinking water 40 mg/kg throughout the experiment. Resveratrol was given orally (1.2 g/kg) three times a week. All treatments alone or in combination were given for three weeks.	To determine the antiproliferative and antitumoral effect of the combination of calcitriol with two phytochemicals, curcumin or resveratrol.	In vitro: The combined treatment presented better antiproliferative properties than treatments alone In vivo: tumor onset, volume and micro-vessel density were significantly reduced in mice co-administered with calcitriol and curcumin Vessel count was also reduced in mice simultaneously treated with calcitriol and resveratrol The concomitant administration of calcitriol with curcumin or resveratrol synergistically promoted anticancer effects in vitro and in vivo in the human mammary tumor cell model.	[74]
Calcitriol alone or with dovitinib	Six-week-old female athymic female nude mice	Calcitriol was intraperitoneally administered 0.25 μg/100 μL each week. Dovitinib was intraperitoneally administered 20 mg/kg twice a week.	To evaluate whether an improved antineoplastic effect could be achieved in vitro and in vivo in TNBC by combining dovitinib, a multi-kinase inhibitor, with calcitriol.	In vitro and in vivo, the drug combination elicited a synergistically improved antiproliferative effect in TNBC-derived cells, which allowed a 7-fold dovitinib dose-reduction.	[22]
**Clinical Trials**					
**Drugs**	**Clinical Trial**	**Doses**	**Aim**	**Results**	**Ref.**
Calcitriol	Phase I (Patients with advanced malignancy)	2 to 10 μg of calcitriol subcutaneously for 4 months.	To determine if a subcutaneous administration of calcitriol can achieve tolerable toxicity in order to ameliorate the hypercalcemia as a major side effect.	The subcutaneous administration led to three pharmacokinetic phases: the initial rapid absorption (Cpmax at two h) of calcitriol from s.c. tissues, a second phase in which plasma calcitriol remained constant for ~6 h, and a third phase starting 8 h after administration in which calcitriol plasma levels declined. The half-life of s.c. calcitriol administration was significantly longer than that reported after oral administration. This study demonstrated that s.c. calcitriol can be administered safely at doses up to 4–5-fold higher than the usual oral dose of 1.5–2.0 μg per day. The MTD for this trial was >5 times the 1.5 μg oral daily dose. No significant antitumor responses were demonstrated in this trial.	[218]
Calcitriol	Phase I trial patients with refractory malignancies	Four weeks of oral, weekly treatment of calcitriol from 0.06–2.8 μg/kg.	To determine the range of escalation doses of calcitriol administrated orally and to establish an ideal dose of it for future evaluations.	The dose of 0.5 microg/kg was selected for future evaluation in Phase II studies.	[219]
Calcitriol/Paclitaxel	Phase I	Calcitriol was given orally for three consecutive days each week at escalating doses, and paclitaxel (80 mg/m^2^) was given intravenously weekly. The starting dose of calcitriol was 4 μg for three consecutive days each week, and the maximum dose administered was 38 μg for three consecutive days each week.	To determine the MTDand pharmacokinetics of calcitriol when administered with paclitaxel in patients with advanced cancer. To evaluate the relationship between calcitriol dose and hypercalcemia.	Calcitriol plasma concentrations of 600 to 1440 pg/mL were achieved. No dose-limiting toxicity occurred in this trial. Despite variability in absorption, very high doses of calcitriol can be safely administered with paclitaxel. No dose-limiting hypercalcemia or other toxicity was observed in patients with cancer who received high doses of calcitriol plus paclitaxel	[220]
Calcitriol	Phase I: Patients were divided into two cohorts: (A) calcitriol + paclitaxel in patients with advanced solid tumors; (B) calcitriol ± dexamethasone in patients with androgen-independent prostate cancer	Oral administration of 12 μg to 21 μg/capsule of calcitriol were tested in 12 patients with advanced solid tumor, while doses from 13 μg to 36 μg of the liquid formulation of calcitriol were tested in 16 patients advanced solid tumor. Cohort A received calcitriol QDx3 (day l–3) + paclitaxel 80 mg/m^2^ on day 3; cohort B received calcitriol alone QDx3 on week 1, and in subsequent weeks, calcitriol QDx3 (days 1–3) and dexamethasone QDx4 (days 0–3). Treatment was continued until disease progression or occurrence of dose-limiting toxicity. Serum calcium, phosphorus, creatinine, BUN, albumin, and glucose were determined weekly.	To determine whether a liquid calcitriol formulation had a more favorable pharmacokinetic profile than a caplet formulation.	There were no differences in Cmax and AUC_0–24h_ between the two formulations. The result of the use of calcitriol in capsule or liquid form was indistinct; however, at some point, the liquid formulation had the disadvantage of causing transient episodes of diarrhea. The use of dexamethasone is based on previous articles where it is shown that this agent decreases 1,25-D3-induced hypercalcemia and enhances 1,25-D3 antitumor activity. The combination with paclitaxel is based on the fact that no dose-limiting hypercalcemia or other toxicity was observed in patients with cancer who received calcitriol plus paclitaxel in a previous study.	[220,221]
High dose formulation of calcitriol (DN-101)	Patients with different adenocarcinomas including prostate, colon, rectum, gastric, squamous cell carcinoma)	Different oral, weekly doses of a high dose of a commercial presentation of calcitriol (DN-101) were given to patients with cancer (15, 30, 45, 60, 75, 90, 105, 135, 165, 210, 270, and 345 μg)	To establish a safe dose for weekly repeat dosing of DN-101. To compare the pharmacokinetic profile of DN-101 and rocaltrol.	Calcium and serum chemistry were monitored every two weeks. In general, DN-101 was very well tolerated on a weekly schedule. However, hypercalcemia was found at 60 μg. Thus, 45 μg is recommended as a safe dose for phase II studies in patients with different adenocarcionamas. Of note, this study did not include patients with BC.	[222]
Calcitriol/Gefitinib	Phase I	Calcitriol was given i.v. over 1 h on weeks 1, 3, and weekly after that. Gefitinib was given at a fixed oral daily dose of 250 mg starting at week 2 (day 8)	To evaluate MTD of this combination.	High doses of weekly i.v. calcitriol can be administered safely in combination with gefitinib. The MTD for calcitriol was 74 μg. The study design did not permit the evaluation of the effects of calcitriol on gefitinib.	[178]
Calcitriol/Gefitinib/Dexamethazone	Phase I	A fixed oral dose of dexamethasone of 4 mg/day was given. Calcitriol was administered i.v. over 1 h on weeks 1, 3, and weekly after that. The starting calcitriol dose level was 57 μg, and escalation occurred in cohorts of three patients until the MTD was defined. Gefitinib was given at a fixed oral daily dose of 250 mg starting at week 2 (day 8).	To determine the MTD of i.v. calcitriol administered in combination with a fixed oral dose of dexamethasone and gefitinib in patients with refractory solid tumors including, colorectal, head and neck, prostate, sarcoma, breast, stomach, non-small cell lung cancer, gastrointestinal stromal tumor and urachal.	The addition of a low dose of dexamethasone allowed the safe escalation of calcitriol to the MTD of 125 μg/week. However, no antitumor activity was observed in patients with different solid tumors. Of note, the study included only one patient with BC.	[179]
Alendronate and calcitriol	Double-blind, prospective, placebo-controlled 24-week trial with a daily combination of alendronate and calcitriol in Hormone- positive patients with early BC.	Daily, oral administration of Maxmarvil^®®^ (5 mg of alendronate and 0.5 μg of calcitriol) for 24 weeks.	To determine whether a lower dosage of alendronate in oral form combined with calcitriol can effectively manage AI-induced bone loss.	The study demonstrated that a combination of 5 mg alendronate and 0.5 μg calcitriol is effective to prevent bone loss due to aromatase inhibitor regimen in post-menopausal women with early BC.	[230]
Calcitriol	Post-menopausal patients (33) with operable BC, without distant metastasis.	Oral administration of 0.50 μg/day (Rocaltrol).	To evaluate the antitumor effects of a short period of VD3 supplementation.	The blood analysis demonstrated that 87.5% of patients had a deficiency of calcitriol, as determined by calcidiol serum levels. Interestingly in paired samples collected before and after calcitriol supplementation, no differences were detected in calcidiol serum concentration. Data from pre- and post- calcitriol supplementation showed a modest reduction, around 35%, of Ki67 expression. Enriched molecular probes demonstrated that target genes of calcitriol were not modulated after the calcitriol supplementation.	[227]

## 11. Conclusions

Several studies have addressed the effects of calcitriol and its analogs in BC, showing different outcomes. However, many support that the combination of calcitriol with conventional BC drugs or with endocrine therapy provide potential therapeutic advantages, due to the proprieties that VD3 compounds exert in combination, such as increasing growth arrest, apoptosis, and anti-inflammatory and antiproliferative effects. Additional outcomes include the regulation of ER expression by VD3 metabolites, which play a role in enhancing antitumor activity of the therapies in both ER-positive and ER-negative cells; re-establishing antiestrogens response, reducing adverse events such as loss of bone, genitourinary atrophy and musculoskeletal symptoms, inhibiting estradiol-stimulated proliferation, suppressing aromatase expression, and disruption of estrogen-dependent signaling.

Although various clinical studies have focused on administering calcitriol in patients with BC to find the MTD of this compound alone or in different combination schemes, these studies have not specifically evaluated their antitumor activity or in many of them, a clear effect was not reported. Other works suggested that VD3 supplementation in terms of prevention of BC risk may be overestimated [251]. However, a great number of preclinical studies have demonstrated a clear antitumor effect of calcitriol or its analogs in different hormone-responsive or non-hormone-responsive BC models. The focus should be on avoiding its calcemic effects and maintaining adequate calcium plasma concentrations in vivo, which can be achieved by intermittent administration of calcitriol. In addition, other factors should be taken into consideration in clinical and preclinical studies concerning calcitriol antitumoral effects in patients with BC, including different populations, VDR polymorphisms, and the status of the enzymes involved in VD3 activation, such as CYP2R1, CYP27A1, CYP27B1, and CYP24A1 [224], all this due to the presence of mutations in these enzymatic components [252] or pharmacological interactions [253].

Interestingly, the antitumor therapy that has recently gained clinical relevance focuses on new routes of direct administration of antiproliferative agents intratumorally [254,255]. In this regard, few studies at the preclinical level have evaluated new forms of calcitriol release into the tumors [256,257]. Thus, we encourage the use of novel routes of calcitriol administration that could allow reaching significant intratumoral concentrations of this hormone, avoiding its main side effects related to calcium. In addition, the use of different combinations of calcitriol with other agents provides potential advantages, increasing the therapeutic effect, reducing the doses of specific drugs in combined schemes, and decreasing the undesirable side effects and drug resistance.

## Abbreviations

15-PGDH	15-hydroxyprostaglandin dehydrogenase
AIs	Aromatase inhibitors
AKT	Protein kinase B
ALDH1 ATRA	Aldehyde dehydrogenase 1 All-trans-retinoic acid
BC	Breast cancer
Bcl-2	B cell CLL/lymphoma-2
BCSC	Breast cancer stem cells
CCND1	Cyclin D1
CD	Cluster of differentiation
CDK	Cyclin-dependent kinases
COX	Cyclooxygenase
CSCs DBP	Cancer stem cells Vitamin D binding protein
DNMT	DNA methyltransferase
EAG1	*Ether-a-go-go*-1 potassium channel
EGFR	Epidermal growth factor receptor
EMT	Epithelial-mesenchymal transition
EP	Prostaglandin E receptor
ER	Estrogen receptor
ERK	Extracellular signal-regulated kinases
FP	Prostaglandin F receptor
HDAC	Histone deacetylases
HDACI	Histone deacetylase inhibitors
HER2	Epidermal growth factor receptor type 2
HERG	Human EAG related genes
i.v.	Intravenous
IGF-1	Insulin-like growth factor 1
IGFBP-3	Insulin-like growth factor binding protein 3
JAK	Janus kinase
MAPK	Mitogen-activated protein kinase
MDM2	Murine double minute 2
MMP	Metalloproteinase
MTD	Maximum tolerated dose
NFκβ	Nuclear factor-kappa beta
NMU	N-methylnitrosourea
NSAIDs	Nonsteroidal anti-inflammatory drugs
OVX	Ovariectomized
PARP	Poly (ADP-ribose) polymerase
PI3K	Phosphatidylinositol 3-kinase
PLA	Phospholipase A2
PR	Progesterone receptor
QDR	Days a week
RARs	Retinoid acid receptors
RAREs	Retinoic-Acid-Response-Elements
ROS	Reactive oxygen species
RXR	Retinoid-X receptor
SERD	Selective estrogen receptor down-
SERM	Selective estrogen receptor modulator
STAT	Activator of transcription
TGF-β1	Transforming growth factor-beta 1
TIL	Tumor-infiltrating lymphocytes
TKI	Tyrosine kinase inhibitors
TNBC	Triple-negative breast cancer
TNFα	Tumor necrosis factor alpha
TSA	Trichostatin A
TSP-1	Thrombospondin 1
VD	Vitamin D
VD3	Vitamin D_3_
VDR	Vitamin D receptor
VDREs	Vitamin D response elements
VEGF	Vascular endothelial growth factor
